# Appropriateness of bone density measurement in Switzerland: a cross-sectional study

**DOI:** 10.1186/s12889-018-5305-0

**Published:** 2018-04-02

**Authors:** Stefan Zechmann, Nathalie Scherz, Oliver Reich, Beat Brüngger, Oliver Senn, Thomas Rosemann, Stefan Neuner-Jehle

**Affiliations:** 1 Institute of Primary Care, University of Zurich, Pestalozzistrasse 24, 8091 Zurich, Switzerland; 2 Department of Health Sciences, Helsana Group, Zurich, Switzerland

**Keywords:** Appropriateness, Bone density measurement, DXA, Screening, Osteoporosis, Switzerland

## Abstract

**Background:**

According to the WHO, osteoporosis is one of the most important non- communicable diseases worldwide. Different screening procedures are controversially discussed, especially concerning the concomitant issues of overdiagnosis and harm caused by inappropriate Dual X-ray Absorptiometry (DXA). The aim of this study was to evaluate the frequency and appropriateness of DXA as screening measure in Switzerland considering individual risk factors and to evaluate covariates independently associated with potentially inappropriate DXA screening.

**Methods:**

Retrospective cross-sectional study using insurance claim data of 2013. Among all patients with DXA screening, women < 65 and men < 70 years without osteoporosis or risk factors for osteoporosis were defined as receiving potentially inappropriate DXA. Statistics included descriptive measures and multivariable regressions to estimate associations of relevant covariates with potentially inappropriate DXA screening.

**Results:**

Of 1,131,092 patients, 552,973 were eligible. Among those 2637 of 10,000 (26.4%) underwent potentially inappropriate DXA screening. Female sex (Odds ratio 6.47, CI 6.41–6.54) and higher age showed the strongest association with any DXA screening.

Female gender (Odds ratio 1.84, CI 1.49–2.26) and an income among the highest 5% (Odds ratio 1.40, CI 1.01–1.98) were significantly positively associated with potentially inappropriate DXA screening, number of chronic conditions (Odds ratio 0.67, CI 0.65–0.70) and living in the central region of Switzerland (Odds ratio 0.67, CI 0.48–0.95) negatively.

**Conclusion:**

One out of four DXAs for screening purpose is potentially inappropriate. Stakeholders of osteoporosis screening campaigns should focus on providing more detailed information on appropriateness of DXA screening indications (e.g. age thresholds) in order to avoid DXA overuse.

**Electronic supplementary material:**

The online version of this article (10.1186/s12889-018-5305-0) contains supplementary material, which is available to authorized users.

## Background

According to the WHO osteoporosis is one of the most important non-communicable diseases worldwide [1] with a high burden of disease [2–5]. In Switzerland, one out of three women at the age of 85 is at risk for an osteoporotic fracture within ten years [6]. Efficient drugs, which are able to prevent osteoporotic fractures, are available [7–9]. Thus identifying patients likely to benefit from such a therapy is of crucial importance. Besides risk calculators based on individual behavior characteristics [10, 11], the standard screening method is Dual X-ray Absorptiometry (DXA) [12]. Its evidence is controversially discussed at present, especially concerning the issues of overdiagnosis and harm resulting from inappropriate DXA screening [13–18]: DXA as a single procedure does not harm patients directly, as the amount of radiation applied is almost negligible. Nevertheless harmful effects of radiation can sum up. The real harm of inappropriate DXA consists in potential additional diagnostic procedures (e.g. repeated DXA’s) as well as concomitant treatment due to diagnostically significant but clinically irrelevant DXA results. DXA measurements are inherently uncertain in relation to fracture risk prediction [19]. If treatment is initiated based on DXA results alone, e.g. in asymptomatic persons regardless of risk factors, it is potentially inappropriate and can cause potentially severe side effects as well as avoidable costs.

Current knowledge on the use of DXA screening in Switzerland is scarce. Therefore, the aim of this study was to evaluate the frequency and appropriateness of DXA as a screening measure in Switzerland, considering individual risk factors, and to evaluate covariates independently associated with potentially inappropriate DXA screening.

## Methods

### Study design and setting

**Fig. 1:**
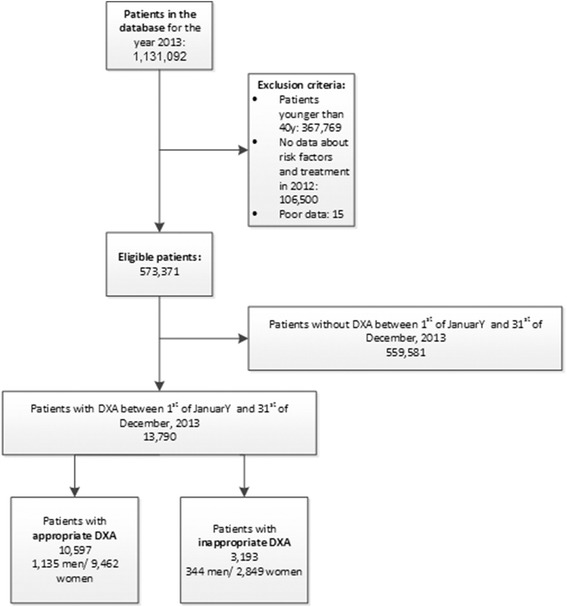
Patient inclusion flowchart. This figure shows individual reasons why patients were excluded and the amount of patients finally included in the study

Retrospective cross-sectional analysis using insurance claims data from the largest insurance company in Switzerland (Helsana). Data covered in- and outpatient health care in Switzerland, where all residents are have mandatory health insurance provided by private health insurance companies (details on various insurance models see below). The study population was extracted from a dataset of 1,131,092 patients across Switzerland, representing a sample of approximately 14% of the Swiss population (see Fig. 1).

### Subjects and data collection

#### Inclusion criteria

All health claims submitted to Helsana for reimbursement of health care provided between January 1st and December 31st 2013 were considered and included following codes:

- Outpatient setting: Outpatient care fees-for-services positions (Tarmed) and Anatomical Therapeutic Chemical (ATC) Classification System for drugs.
- Inpatient setting: Diagnosis Related Groups (DRG), International Classification of Diseases (ICD) and Swiss classification of operations (CHOP) for surgical interventions. An additional file shows this in more detail [see Additional file 1].

#### Exclusion criteria

- No data on area of living
- No data on risk factors and treatment of osteoporosis in 2012
- No data on treatment of osteoporosis in 2014
- Previous use of antiresorptive medication (in this condition DXA might be used for monitoring of treatment and not for screening purpose)
- Diagnosis of osteoporosis
- Age below 40 years

### Definition of osteoporosis and risk factors for osteoporosis

Osteoporosis was defined by diagnostic code (ICD, DRG) or use of antiresorptive medication (ATC). Risk factors for osteoporosis were defined according to the FRAX® score [11] and according to recommendations of the Swiss Association against Osteoporosis (SVGO) [20] (Table 1).

**Table 1:** Definitions of osteoporosis and risk factors of osteoporosis

Characteristics	Coding System	Indicator
Osteoporosis ^a^	Using ICD, ATC codes	Diagnostic code (ICD, DRG), antiresorptive medication (ATC)
Risk factors for osteoporosis according to FRAX® score^a^ [11]	Using ICD, DRG, CHOP, Tarmed and ATC codes	Steroid medication, fracture in typical location (distal radius fracture, proximal humerus fracture, vertebral fracture, femur fracture) rheumatoid arthritis, insulin dependent diabetes (as a proxy variable for type I diabetes), osteogenesis imperfecta, hyperthyroidism, hypogonadism, chronic liver disease, nicotine and alcohol abuses, malnutrition, underweight or malabsorption (including gastric by-pass and inflammatory bowel disease)
Risk factors for osteoporosis according to Swiss Association against Osteoporosis (SVGO) ^a^	Using ICD, DRG, CHOP, Tarmed and ATC codes	Hyperparathyroidism, hypothyroidism, asthma or COPD, multiple myeloma, antiepileptic drugs and anti-HIV drugs

Definition of clinical characteristics and medication; ^a^ Data 6 months prior to DXA screening were taken into account

An additional file shows this in more detail [see Additional file 1].

### Variables

#### Socio-demographic characteristics

- Age
- Gender
- Chronic conditions: According to the pharmaceutical cost group (PCG) model by Huber et al. [21]. We additionally defined a minimum of three prescriptions per year as precondition for the following chronic conditions: acid related disorders, pain, psychological disorders (sleep disorder, depression) and rheumatological conditions.
- Area of living: Typology of Swiss communities defined by the Swiss Federal Statistical Office into ‘urban’ (‘central city’, ‘isolated city’, ‘agglomeration’), and ‘rural’ (‘rural areas’) (data originates from “Gemeindetypologie - Bundesamt für Statistik” 2003)
- Density of DXA facilities: The number of DXA facilities per canton was adjusted to the number of residents per canton and then categorized into four quartiles (data originates from “Bewilligungstatistik - Bundesamt für Gesundheit” 2016)

#### Socio-economic characteristics

- Socio-economic status using income levels per corresponding zip code as a proxy [22]. Income level categorized into lowest 5%, middle 90%, and highest 5%.
- Insurance Status:
  - Supplementary private hospital insurance
  - Deductibles ≥ or < 1000 Swiss francs (CHF); High deductible class, e.g. 1000, 1500, 2000, or 2500 CHF as compared to the standard deductible of 300 or 500 CHF
  - Managed care: We defined health plans with capitation, family doctor models, or telemedicine models as managed care models.

### Analysis

First we calculated number and proportion of patients receiving any DXA screening. In a second step, number and proportion of patients receiving potentially inappropriate respectively appropriate DXA screening were calculated.

Any DXA screening performed on women < 65 and on men < 70 years without any risk factors for osteoporosis was defined as potentially inappropriate. For presentation of results, data was categorized by 5-year age strata, gender and existence of risk factors.

DXA screening performed on women > 65 and on men > 70 years or DXA screening performed on women < 65 and on men < 70 years *with* risk factors for osteoporosis was defined as appropriate DXA screening. For presentation, data was categorized by 5-year age strata, gender and existence of risk factors.

Furthermore, we analyzed independent covariates for receiving any DXA screening as well as independent covariates for receiving potentially inappropriate DXA screening.

We used descriptive statistics to provide a general profile of the study population. For continuous variables, we used means with standard deviations (SD) or median and interquartile ranges (IQR), for categorical variables counts and percentages. We used Kruskal-Wallis, Fisher exact and Chi-Square tests as applicable to compare the group with and without any DXA, respectively. We categorized cases by gender and 5-year strata of age.

We performed multivariable regression analysis to estimate the effect of any of the covariates age, gender, health insurance coverage, region density of DXA facilities, income level, number of chronic conditions, risk factors and known osteoporosis on the patient’s probability of having any DXA. In the model for the patient’s probability of having a potentially inappropriate DXA, we included the covariates age, gender, health insurance coverage, region, income level, number of chronic conditions and density of DXA facilities. For these multivariable models, we used logistic regression.

## Results

From our database consisting of 1,131,092 patients, 552,973 were finally included for analysis (See Fig. 1). The majority of patients was excluded due to age below 40 years (365,511), missing data on risk factors and treatment of osteoporosis in 2012 (106,500), as well as due to missing data on treatment of osteoporosis in 2014 (83,437).

Included patients had a median age of 60 (IQR 22.0) years, 292,377 (52.9%) were female, 424,875 (76.8%) were living in urban areas and had a median of 1 (IQR 3.0) chronic conditions (see Table 2).

**Table 2:** Demographic baseline data

Covariates	Total	No DXA	Any DXA	*p*-Value	Test
n	552,973	542,973 (98.2%)	10,000 (1.8%)		
Age	60.0 (22.0)	60.0 (21.0)	66.0 (16.0)	< 2.2e-16 ***	‘
Female sex	292,377 (52.9%)	283,493 (52.2%)	8884 (88.8%)	< 2.2e-16 ***	“
Managed care	249,826 (45.2%)	245,628 (45.2%)	4198 (42.0%)	8.52e-11 ***	“
Deductible ≥ CHF 1000	143,295 (25.9%)	142,212 (26.2%)	1083 (10.8%)	< 2.2e-16 ***	“
Supplementary hospital insurance	127,080 (23.0%)	123,689 (22.8%)	3391 (33.9%)	< 2.2e-16 ***	“
Region				< 2.2e-16 ***	““
Midland	110,347 (20.0%)	108,473 (20.0%)	1874 (18.7%)		
Northwest	76,568 (13.8%)	75,141 (13.8%)	1427 (14.3%)		
East	78,285 (14.2%)	76,914 (14.2%)	1371 (13.7%)		
Lake Geneva	70,177 (12.7%)	68,800 (12.7%)	1377 (13.8%)		
Ticino	40,729 (7.4%)	39,915 (7.4%)	814 (8.1%)		
Central	48,871 (8.8%)	48,225 (8.9%)	646 (6.5%)		
Zurich	127,996 (23.1%)	125,505 (23.1%)	2491 (24.9%)		
Urban area	424,875 (76.8%)	416,774 (76.8%)	8101 (81.0%)	< 2.2e-16 ***	“
Density DXA facilities				3.18e-13 ***	““
1. quartile	146,876 (26.6%)	144,549 (26.6%)	2327 (23.3%)		
2. quartile	158,878 (28.7%)	155,932 (28.7%)	2946 (29.5%)		
3. quartile	156,620 (28.3%)	153,514 (28.3%)	3106 (31.1%)		
4. quartile	90,599 (16.4%)	88,978 (16.4%)	1621 (16.2%)		
Purchasing power				3.39e-07 ***	““
middle	498,052 (90.1%)	489,136 (90.1%)	8916 (89.2%)		
lowest	28,221 (5.1%)	27,744 (5.1%)	477 (4.8%)		
highest	26,700 (4.8%)	26,093 (4.8%)	607 (6.1%)		
Use of antidepressive drugs	59,602 (10.8%)	57,984 (10.7%)	1618 (16.2%)	< 2.2e-16 ***	“
Risk factor: History of fracture	1874 (0.3%)	1673 (0.3%)	201 (2.0%)	< 2.2e-16 ***	“
Risk factor: Osteogenesis imperfecta	1 (0.0%)	1 (0.0%)	0 (0.0%)	1	“
Risk factor: Hypogonadism	3562 (0.6%)	2989 (0.6%)	573 (5.7%)	< 2.2e-16 ***	“
Risk factor (SVGO): Hyperparathyroidism	341 (0.1%)	317 (0.1%)	24 (0.2%)	2.53e-08 ***	“
Risk factor: Hyperthyroidism	1171 (0.2%)	1136 (0.2%)	35 (0.4%)	0.005611 **	“
Risk factor (SVGO): Hypothyroidism	25,595 (4.6%)	24,621 (4.5%)	974 (9.7%)	< 2.2e-16 ***	“
Risk factor: Malnutrition	2270 (0.4%)	2207 (0.4%)	63 (0.6%)	0.001148 **	“
Risk factor: Steroid therapy	45,443 (8.2%)	43,206 (8.0%)	2237 (22.4%)	< 2.2e-16 ***	“
Risk factor: Inflammatory bowel disease	4291 (0.8%)	3990 (0.7%)	301 (3.0%)	< 2.2e-16 ***	“
Risk factor: Rheumatoid arthritis	4900 (0.9%)	4401 (0.8%)	499 (5.0%)	< 2.2e-16 ***	“
Risk factor: Insulin dependent diabetes	11,399 (2.1%)	11,210 (2.1%)	189 (1.9%)	0.241	“
Risk factor: Chronic liver disease	507 (0.1%)	489 (0.1%)	18 (0.2%)	0.006869 **	“
Risk factor: Nicotine abuse	893 (0.2%)	871 (0.2%)	22 (0.2%)	0.1641	“
Risk factor: Alcohol abuse	1435 (0.3%)	1400 (0.3%)	35 (0.4%)	0.07417	“
Risk factor (SVGO): Emerging risk factors	38,633 (7.0%)	37,539 (6.9%)	1094 (10.9%)	< 2.2e-16 ***	“
Number of chronic conditions	1.0 (3.0)	1.0 (3.0)	2.0 (3.0)	< 2.2e-16 ***	‘

Number and proportion of covariates of patients with any DXA screening and with no DXA screening respectively. Abbreviations: “Total” = all included patients, “Any DXA” = all patients who received any DXA screening, “No DXA” = all patients receiving no DXA screening. Significance levels are marked accordingly: *** ≤ 0.001, ** = 0.001–0.01, * = 0.01–0.05, empty = 0.05–1. ‘= Kruskal-Wallis test, “= Fisher exact test, ““= Chi-Square test

### Proportion of patients with *any* DXA screening

10,000 (1.8%) of all patients (552,973) included in this study received any DXA screening. Median age of patients with any DXA screening was 66 (IQR 16.0) years, 8884 (88.8%) were female, 8101 (81.0%) lived in urban areas and had a median of 2 (IQR 3.0) chronic conditions. The age of patients with any DXA was significantly higher than the age of patients with no DXA (mean age 66.0 compared to 60.0). The proportion of female sex among patients with any DXA was significantly higher compared to those patients with no DXA (88.8% vs. 52.2%). Also most other covariates differed significantly between patients with any DXA and patients without (see Table 2).

### Proportion of patients with *potentially inappropriate* DXA screening

2637 (26.4%) of patients with DXA (10,000) received a potentially inappropriate DXA screening. 2378 (90.2%) were female and 259 (9.8%) were male. By definition, only patients in the age groups < 65 years in women and in the age groups < 70 years in men were potentially affected (see Table 3).

**Table 3:** Appropriateness of DXA

	Men			Women		
	n of patients with DXA without riskfactor	n of patients with DXA with ≥ 1 risk factor	N of patients in the sample	n of patients with DXA without risk factor	n of patients with DXA with ≥ 1 risk factor	N of patients in the sample
40–44	16	24	32,081	95	70	31,480
45–49	27	50	35,421	206	168	34,998
50–54	39	67	34,311	471	296	34,558
55–59	40	85	31,922	715	443	32,743
60–64	62	70	30,005	891	548	32,141
65–69	75	109	29,949	919	652	32,112
70–74	78	103	24,421	774	651	28,148
75–79	44	85	18,927	534	530	24,150
80–84	44	58	13,477	327	313	20,360
85–89	19	15	7124	120	122	13,878
≥ 90	2	4	2958	19	20	7809
Sum	446	670	260,596	5071	3813	292,377

Number of patients with potentially inappropriate respectively appropriate DXA screening stratified by 5-year strata of age and sex

### Independent covariates associated with *any* DXA screening

**Fig. 2:**
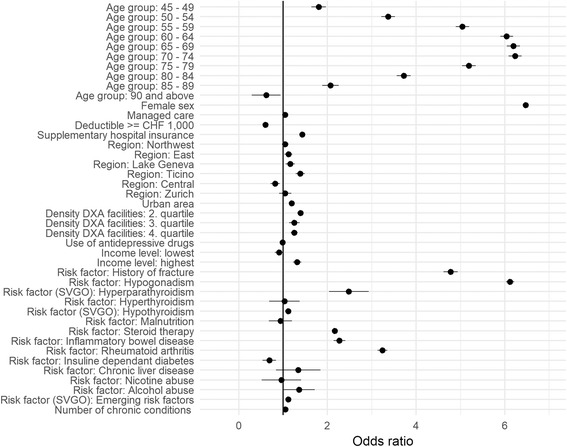
Multivariable model of any DXA. Multivariable analysis of socio-demographic, socio-economic and clinical covariates of patients receiving any DXA

The majority of investigated covariates were significantly (*p* < 0.002) associated with any DXA screening (see Fig. 2). Following covariates showed the greatest effect: Female sex (Odds ratio 6.47, CI 6.41–6.54), age group 70–74 (Odds ratio 6.23, CI 6.08–6.38), age group 65–69 (Odds ratio 6.19, CI 6.04–6.34), age group 75–79 (Odds ratio 5.18, CI 5.03–5.34), age group 55–59 (Odds ratio 5.04, CI 4.89–5.19) as well as the risk factors hypogonadism (Odds ratio 6.12, CI 6.02–6.22), history of fracture (Odds ratio 4.78, CI 4.62–4.94) and rheumatoid arthritis (Odds ratio 3.24, CI 3.13–3.35). Details see Fig. 2 and Additional file 2.

#### Independent covariates associated with *potentially inappropriate* DXA screening

**Fig. 3:**
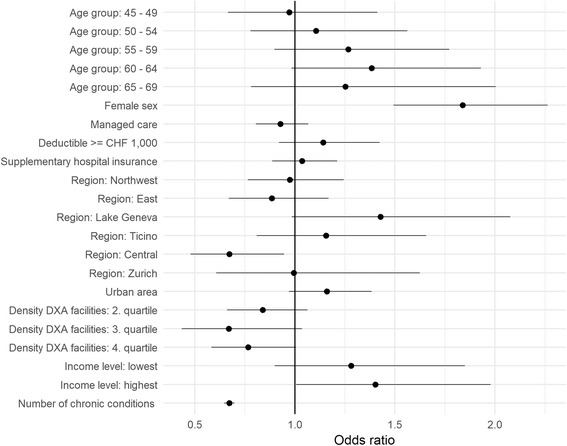
Multivariable model of potentially inappropriate DXA. Multivariable analysis of age, gender, socio-demographic, socio-economic and clinical covariates of patients receiving a potentially inappropriate DXA

Female sex (Odds ratio 1.84, CI 1.49–2.26), income level among the highest 5% (Odds ratio 1.40, CI 1.01–1.98), number of chronic conditions (Odds ratio 0.67, CI 0.65–0.70) and living in the central region of Switzerland (Odds ratio 0.67, CI 0.48–0.95) were significantly (*p* < 0.05) associated with potentially inappropriate DXA screening. All other covariates were not significantly associated (Details see Fig. 3 and Additional file 3).

## Discussion

In this cross-sectional study, 2637 patients (26.4% of all 10,000 patients with any DXA screening) received a potentially inappropriate DXA. Female gender and income level among the highest 5%, were significantly positively associated with potentially inappropriate DXA screening, number of chronic conditions and living in the central region of Switzerland negatively.

Overall 10,000 patients (1.8% of our total study population) received any DXA screening, 8884 (3.0%) of all women and 1116 (0.4%) of all men in 2013. Previous studies found higher proportions of patients with any DXA, e.g. 20% for women and 3 to 4% for men [13, 23, 24]. Comparisons between these studies seem difficult due to differences in populations, settings and time periods. Female gender, age and the majority of established risk factors for osteoporosis were significantly associated with any DXA screening, corresponding with the literature [13, 18, 23, 25, 26]. This finding confirms that the set of covariates chosen in our study is valid for discriminating between patients at risk for osteoporosis and patients not at risk.

In contrast to our finding that 26.8% of all women and 23.2% of all men received a potentially inappropriate DXA, previous studies reported 10 to 20% of inappropriate DXA among women and 16% among men [16]. Frost et al. found that one out of three patients with DXA did not have any risk factor for osteoporosis, while one out of five patients with DXA did not have any risk factor when family history and falls are included [18].

The number of potentially inappropriate DXA increased continuously until the age of 65 years (e.g. almost doubling with the age of 60–65 compared to 50–55). Female gender and a higher age are the two risk factors best known to patients [27]. This specific subgroup might ask more actively for DXA screening due to their higher risk awareness. This might be a result of public campaigns successfully fostering awareness of osteoporosis among this specific subgroup, at least in high-income countries like Switzerland [28]. Unfortunately, most public campaigns fail to provide information on *inappropriate* DXA screening indications (e.g. younger age). Gender differences in screening programs in general, as well as in osteoporosis screening programs, have been observed before [29–35]. Besides inherent gender differences, lack of campaigns promoting health specifically for men might be a major contributor to this finding [36, 37]. Amarnath et al. argue that some physicians still use DXA as a screening tool for all menopausal women regardless of age or other risk factors [13].

An income level among the highest 5% was significantly associated with inappropriate DXA screening as well. A higher level of income might be a proxy for higher education [25]. Health campaigns usually reach higher educated people rather than lower educated, leading to a higher risk awareness and resulting in higher demand for screening among this subgroup. Amarnath et al. found underuse of other screening procedures in patients with lower education level, confirming our finding [13].

The number of chronic conditions commonly used as a proxy for an elderly, multimorbid population was significantly negatively associated with a potentially inappropriate DXA screening. As multimorbidity is associated with age and frailty, this population is more prone to osteoporosis, and thus more frequently and more appropriately transferred to DXA screening.

Variations between regions might be due to differences in needs and attitudes of patients or physicians living in these regions. Furthermore, differences in physician density and therefore unequal access to medical supply could be an additional reason. Born et al. reported similar findings for the central and Lake Geneva region of Switzerland as well [25].

Living in a canton with a higher density of DXA facilities led to a higher proportion of *any* DXA screening. Rubin et al similarly reported a higher density of DXA facilities as a trigger for a higher use of DXA [16]. This findings undermined the economic model that higher supply is likely to trigger higher demand [38]. Remarkably, there was no significant association between a higher density of DXA facilities and potentially inappropriate DXA, therefore not directly contributing to overuse.

We found a significant positive association with a deductible ≥1000CHF and supplementary hospital insurance with any DXA. However, there was no significant association of these covariates with potentially inappropriate DXA. As discussed above a supplementary hospital insurance is a possible proxy for a higher income level and thus a higher education leading to a higher demand for DXA screening [25, 39].

Finally, overuse of DXA may be triggered by physicians’ and patients’ belief that the potential benefit of DXA is weighing out its minimal risk of harm, even in the absence of risk factors for osteoporosis, regardless of the risk of overdiagnosis and overtreatment [13, 40, 41].

### Strengths

A major strength of this study is the large and representative sample size (14% of the Swiss population). Many studies investigating proportions and covariates of DXA screening were based on smaller samples [16, 18, 25, 42].

We focused on DXA for screening intentions only, while most studies did not differentiate between reasons for performing DXA (screening, diagnosis, or monitoring of medical treatment). Hence, we were able to avoid misclassification bias concerning the indication for a DXA.

We tested a set of clinically reasonable covariates, which reasonability was confirmed by the findings of our regression analysis on covariates.

### Limitations

In our database, important risk factors such as family or patient history were not available (a limitation which a majority of comparable studies had to face as well). These circumstances might have led to some misclassification bias. Frost et al. reported that 10.3% and 15.1% of all patients receiving a DXA had a family history of hip fracture and family history of osteoporosis respectively.

The use of data of 1 year (2013) only restricts the comparability to other studies and is therefore a limitation.

### Further research and recommendations

Attitudes and beliefs concerning DXA screening among healthcare providers as well as patients might be interesting aspects to explore by qualitative or mixed methods research. The results of this research might facilitate implementation of more appropriate DXA screening.

As discussed above, further initiatives and campaigns should not only foster awareness, but should also focus on correct information transfer concerning screening indication and benefits.

Additionally a two-step screening process consisting of risk assessment using cost-effective validated tools followed by DXA could be beneficial [43–46].

## Conclusion

One out of four DXAs for screening purpose is potentially inappropriate. Stakeholders of osteoporosis screening campaigns should focus on providing more detailed information on appropriateness of DXA screening indications (e.g. age thresholds) in order to avoid DXA overuse.

## Additional files

Additional file 1: “Definition of variables”. In this file you can see in detail which coding was used for diagnosis and risk factors. (PDF 76 kb)

Additional file 2: “Multivariable model of any DXA”. In this file we present the same data as presented in Fig. 2 (multivariable analysis of socio-demographic, socio-economic and clinical covariates in patients with any DXA), but in tabular form including estimate (Odds ratio), confidence intervals and *p*-value. Significance levels are marked accordingly: *** ≤ 0.001, ** = 0.001–0.01, * = 0.01–0.05, empty = 0.05–1. (XLS 31 kb)

Additional file 3: “Multivariable model of potentially inappropriate DXA”. In this file we present the same data as presented in Fig. 2 (multivariable analysis of age, sex, socio-demographic, socio-economic and clinical covariates in patients with a potentially inappropriate DXA), but in tabular form including estimate (Odds ratio), confidence intervals and p-value. Significance levels are marked accordingly: *** ≤ 0.001, ** = 0.001–0.01, * = 0.01–0.05, empty = 0.05–1. (XLS 29 kb)

## Abbreviations

ATC: Anatomical Therapeutic Chemical (Classification System for drugs)
CHF: Swiss francs
CHOP: Swiss classification of operations
DRG: Diagnosis Related Groups
DXA: Dual X-ray Absorptiometry
ICD: International Classification of Diseases
IQR: Interquartile range
PCG: Pharmaceutical cost group
SD: Standard deviations
SVGO: Swiss Association against Osteoporosis
Tarmed: Outpatient care fees-for-services positions
