# Supplementary material for: Appropriateness of bone density measurement in Switzerland: a cross-sectional study
Source: BMC Public Health. 2018 Apr 2;18:423. doi: 10.1186/s12889-018-5305-0 (PMC5879564; doi:10.1186/s12889-018-5305-0)
Supplement: Supplementary file 1 — “Definition of variables”. In this file you can see in detail which coding was used for diagnosis and risk factors. (PDF 76 kb) [file 12889_2018_5305_MOESM1_ESM.pdf]

Headers	Definition	Risk factor	ICD	DRG	CHOP	TARMED
Risk factors for osteoporosis						
Fracture	Distal radius fracture	1	S52.50-59	I74A, I74C	78.13.10, 78.53.10, 79.02, 79.12.10-19, 79.22.10, 79.32.10	24.2410, 24.2450, 24.2480
	Proximal humerus fracture	1	S42.20-29	I75A-B	78.12.10-11, 78.52, 79.01, 79.11.10-19, 79.21.10, 79.31.10-12	24.1110, 24.1120 24.1130, 24.1140, 24.1160
	Vertebral fracture	1	S22.00-1, S32.00-05	I68B-E	03.53, 79.49.2-79.49.38, 81.65-66	06.0220
	Femur fracture	1	S72.0-3	I66A-E	78.55, 79.05, 79.15, 79.25, 79.35.00-12	24.5010, 24.5020, 24.5030, 24.5040
Osteogenesis imperfecta	Osteogenesis imperfecta	1	Q78.0			
Hypogonadism	Hypogonadism	1	E29.1			24.5060, 24.5070, 24.5080, 24.5090, 24.5120
	Ovarian insufficiency	1	E28.3	N62A-B		
	Orchidectomy				62.41, 62.42	
	Oophorectomy	1	Z90.72	N04A,C	65.51-64	
	Hormonal suppressive	1				
Hyperparathyroidism	Hyperparathyroidism	3	E21.0-3	K64A,D		
	Parathyroidectomy	3	E89.2	K06A-B	06.81-99	14.023
	Parathyroid-Antagonists	3				
Hyperthyroidism	Hyperthyroidism	1	E05	K64A, D		
	Thyroid antagonists	2				
Hypothyroidism	Hypothyroidism	3	E03	K64A, D		
	Thyroid agonists	3				
	Thyroidectomy	3				14.021

Malabsorption	Malabsorption	1	K90	G46A-C, G48A-C, G50Z, G67A-D		
	Gastric by-Pass	1	Z98.84	G18A-B	44.31-39,	
Underweight	Underweight	1	R63.6	K62A-B		
Malnutrition	Malnutrition	1	E46			
Inflammatory bowel	Crohn's disease	1	K50	G46A-C,		
	Ulcerative colitis	1	K51	G46A-C, G47Z, G48A-C, G64A-C		
	Non specified inflammatory bowel disease	1	K52	G46A-C, G47Z, G48A-C, G64A-C		
	Topical corticosteroid	1				
	Topical aminosalycilate	1				
	Folate-analog metabolite inhibitor	1				
	Purine-analog metabolite inhibitor	1				
	Azathioprin	1				
	Calcineurin-inhibitors	1				
	TNF-alpha inhibitors	1				
Rheumatoid arthritis	Rheumatoid arthritis	1	M06	I69Z		
	(Chronic) steroid therapy	1				
	Selective	1				
	Folate-analog metabolite	1				
	TNF-alpha inhibitors	1				
Insuline dependant diabetes	Type I Diabetes Mellitus	1	E10			
	Insuline	3				
Chronic liver disease	Chronic liver disease	1	K73, K74, K75.4, K75.8, K75.9	H60Z, H63A, H63C		
	Alcoholic liver disease	1	K70			
	Toxic liver disease	1	K71.3, K71.4, K71.5			
	Esophageal varices therapy	1		H40Z	42.33, 42.91	
	Gastric varices therapy	1			44.43.10, 44.43.20, 44.49.10, 44.49.11, 44.49.20, 44.49.21, 44.91, 44.92	

	Esophageal or grastral varices therapy					19.0160, 19.0260, 19.0270, 19.0280, 19.0290
Nicotine abuse	Nicotine abuse	1	F17			
	Vareniclin	1				
	Bupropion	1				
Alcohol abuse	Alcohol abuse	1	F10			
	Alcohol withdrawal	1		V60A-B		
	Disulfiram	1				

Headers	Definition	Risk	ICD	DRG	CHOP	TARMED
Previous osteoporose, therapy and treatment						
Osteoporosis	Osteoporosis		M80, M81	I69		
DXA	DXA					39.2140, 39.2150, 39.2160
DXA precription						Prescription from primary care physician /other provider
Antiresorptive						
	Biphosphonate					
	Denosumab					
	Teriparatide					
	Calcitonin					
	Selective estrogen receptor modulators					
	Strontiumranulat					
